# The application of artificial intelligence assistant to deep learning in teachers' teaching and students' learning processes

**DOI:** 10.3389/fpsyg.2022.929175

**Published:** 2022-08-11

**Authors:** Yi Liu, Lei Chen, Zerui Yao

**Affiliations:** ^1^School of Electronic and Information, Dongguan Polytechnic, Dongguan, China; ^2^Department of Ideological and Political Education, Dongguan Technician College, Dongguan, China; ^3^School of Computer Science and Technology, Dongguan University of Technology, Dongguan, China

**Keywords:** artificial intelligence technology, deep learning, intelligent classroom, teaching model, learning behavior

## Abstract

With the emergence of big data, cloud computing, and other technologies, artificial intelligence (AI) technology has set off a new wave in the field of education. The application of AI technology to deep learning in university teachers' teaching and students' learning processes is an innovative way to promote the quality of teaching and learning. This study proposed the deep learning-based assessment to measure whether students experienced an improvement in terms of their mastery of knowledge, development of abilities, and emotional experiences. It also used comparative analysis of pre-tests and post-tests through online questionnaires to test the results. The impact of technology on teachers' teaching and students' learning processes, identified the problems in the teaching and learning processes in the context of the application of AI technology, and proposed strategies for reforming and optimizing teaching and learning. It recommends the application of software and platforms, such as Waston and Knewton, under the orientation of AI technology to improve efficiency in teaching and learning, optimize course design, and engage students in deep learning. The contribution of this research is that the teaching and learning processes will be enhanced by the use of intelligent and efficient teaching models on the teachers' side and personalized and in-depth learning on the students' side. On the one hand, the findings are helpful for teachers to better grasp the actual conditions of in-class teaching in real time, carry out intelligent lesson preparations, enrich teaching methods, improve teaching efficiency, and achieve personalized and precision teaching. On the other hand, it also provides a space of intelligent support for students with different traits in terms of learning and effectively improves students' innovation ability, ultimately achieving the purpose of “artificial intelligence + education.”

## Introduction

Artificial intelligence (AI) technology refers to intelligent machines that are created by human beings and implemented through computer programs with the capabilities of autonomous perception, cognition, decision-making, learning, execution, and social collaboration, which are manipulated by human beings to achieve a series of operations or tasks that are simple and tedious that human beings do not wish to perform themselves or that human beings cannot perform on their own. Technology enables machines to simulate human intelligence, which lies in humans' cognitive abilities embodied in five dimensions: neurological, psychological, linguistic, thinking, and cultural (Drezewski and Solawa, 2021). The core issues of AI technology consist of constructing the capabilities to reason, comprehend, plan, learn, communicate, perceive, move things, use tools, and manipulate machines that are similar to or even beyond the capabilities of humans (Bin and Mandal, 2019). Currently, there are a large number of tools that apply artificial intelligence, such as searching, mathematical optimization, and logical deduction. There are also many explorations into algorithms based on bionics, cognitive psychology, probability theory, and economics. Driven by new theories and technologies such as mobile web, big data, supercomputing, sensor networks, and brain sciences, the development of AI technology has accelerated, presenting new features such as deep learning, interdisciplinary integration, human–machine collaboration, collective open source intelligence, and autonomous manipulation, which exert a significant and far-reaching impact on economic development, social progress, and international, political, and economic landscape. New technologies such as artificial intelligence, the Internet, big data, cloud computing, virtual reality, and the Internet of Things will further promote socio-economic, ideological, and cultural development, as well as reforms in the field of education and pedagogy (Liu et al., 2022a,b). In the context of “China Education Modernization 2030,” to build a country with extraordinary strengths in higher education in the new era, it has become the mission of the era and values to pursue higher education to embrace modernization, the world, and the future, to be in service of the construction of an innovative country, and to cultivate innovative, multidisciplinary, and application-oriented talents. The deep integration of AI technology and higher education has led to transformations in the role of university teachers. With continual reforms of teaching methods, students' ways of learning are also proliferating (Xi, 2020).

Section Introduction of this article introduces the meaning of AI technology and the background of the research and design of the research. Section Relative work takes the design and production of personal resume in the teaching of the public foundational course “Career Development and Employment Guidance for college students” as an example and analyzes the experimental data from the perspective of AI technology. Section Research method analysis explains the impact of AI technology on teaching and learning. Section Findings and discussion clarifies the measures and suggestions for using AI technology to improve teaching and learning for university teachers and students, respectively.

## Relative work

### AI in education

Nowadays, it has become a trend to apply AI technology to the development of education and pedagogy. With the rapid development and widespread application of AI technology, enhancing the efficiency and intelligence of teaching has become a hot topic for research in education and pedagogy, and a new form of education and pedagogy that is deeply intertwined with AI technology—the intelligent classroom—has gradually taken shape (Liu et al., 2020a,b, 2021; Drezewski and Solawa, 2021). Such deep integration is an effective way of teaching to achieve the combination of “guidance–subject” in the classroom and promote students' deep learning. Students' mastery and application of knowledge, cultivation of abilities, and enhancement of emotional experience are the core and main goals of in-class teaching. By implementing three rounds of action research, this study finds the problems of backward teaching methods, students' weak awareness of independent learning, and problems in teacher–student interactions and human–computer interactions encountered in the process of teaching with existing information technology in universities (Agung and Gaol, 2012; Roberto et al., 2020). This study proposes to build an efficient and intelligent in-class teaching model based on AI technology in the hope that this strategy of reform can promote the growth of teachers and students and enhance the efficiency and quality of teaching and learning.

This study aims to enhance students' deep learning ability through three rounds of action research in teaching and learning. The targets of the action research were first-year college students, and the content of the research was the design and production of personal resume in the public foundational course “Career Development and Employment Guidance for College Students.” The action research divided participants into the experimental class and control class, with 40 students in each class (Pavlicek et al., 2014; Gang et al., 2021). In this study, students' deep learning ability was mainly measured in three aspects, namely, students' mastery of knowledge, development of abilities, and enhancement of emotional experience. Among them, students' mastery of knowledge was improved by the questions in the tests. We classified students' works submitted in each round into five levels according to the SOLO taxonomy and used pre-test and post-test questionnaires to measure students' ability in deep learning. Finally, the students' recognition of in-class teaching was examined through interviews (Quan, 2020; Kuleto et al., 2021).

### Deep learning applications

With the advancement of neural networks, it can help automated inspections to identify defects more effectively. Back Propagation Network (BPN) and even Convolutional Neural Networks (CNN), CNN is a major breakthrough (Fu et al., 2020, 2021a,b; Liu et al., 2020c; Zhang et al., 2021a,b). Almost all networks are based on CNN to improve and develop. The detection network used in this research, YOLO (You Only Look Once) is also developed based on CNN (Richter and Streitferdt, 2018; Cheng et al., 2021; Kuleto et al., 2021; Chen et al., 2022).

#### Convolutional neural networks

**Figure 1:**
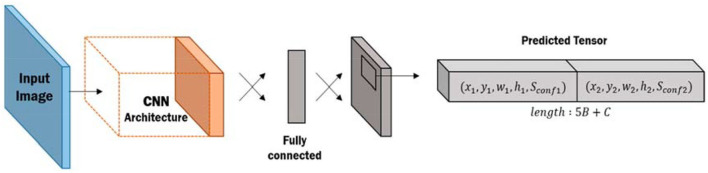
YOLO network architecture diagram.

In the original machine learning, the image needs to be flattened into one-dimensional information and then calculated, but this method will lose the original image characteristics, and the convolutional neural network road is identified by using high-dimensional feature information in the image. For example, humans see birds because they see the beak or the back of the chair. The CNN architecture includes Convolutional layer: Given one or more filters, extract each feature in the image according to its size, and get a feature map as Figure 1. Pooling layer: Retains the important information left after the convolutional layer. Its advantages are that it reduces parameters to speed up calculations (Liu et al., 2020a,b, 2021). If there is a slight change between adjacent pixels, it will have little effect on the output result of the pooling layer and reduce overfitting. Fully connected layer: It flattens the remaining features, performs common neural network operations, and classifies them. CNN goes through a multi-layer convolutional layer and a pooling layer. The convolutional layer is responsible for extracting image features and then leaving important information after the pooling layer. Finally, the fully connected layer performs the final calculation and classification. The proposed convolutional neural network architecture uses high-dimensional feature extraction and has good recognition capabilities (Cheng et al., 2019a; He and Yu, 2019; Huang and Xia, 2020; Chen et al., 2021).

#### YOLO

**Figure 2:**
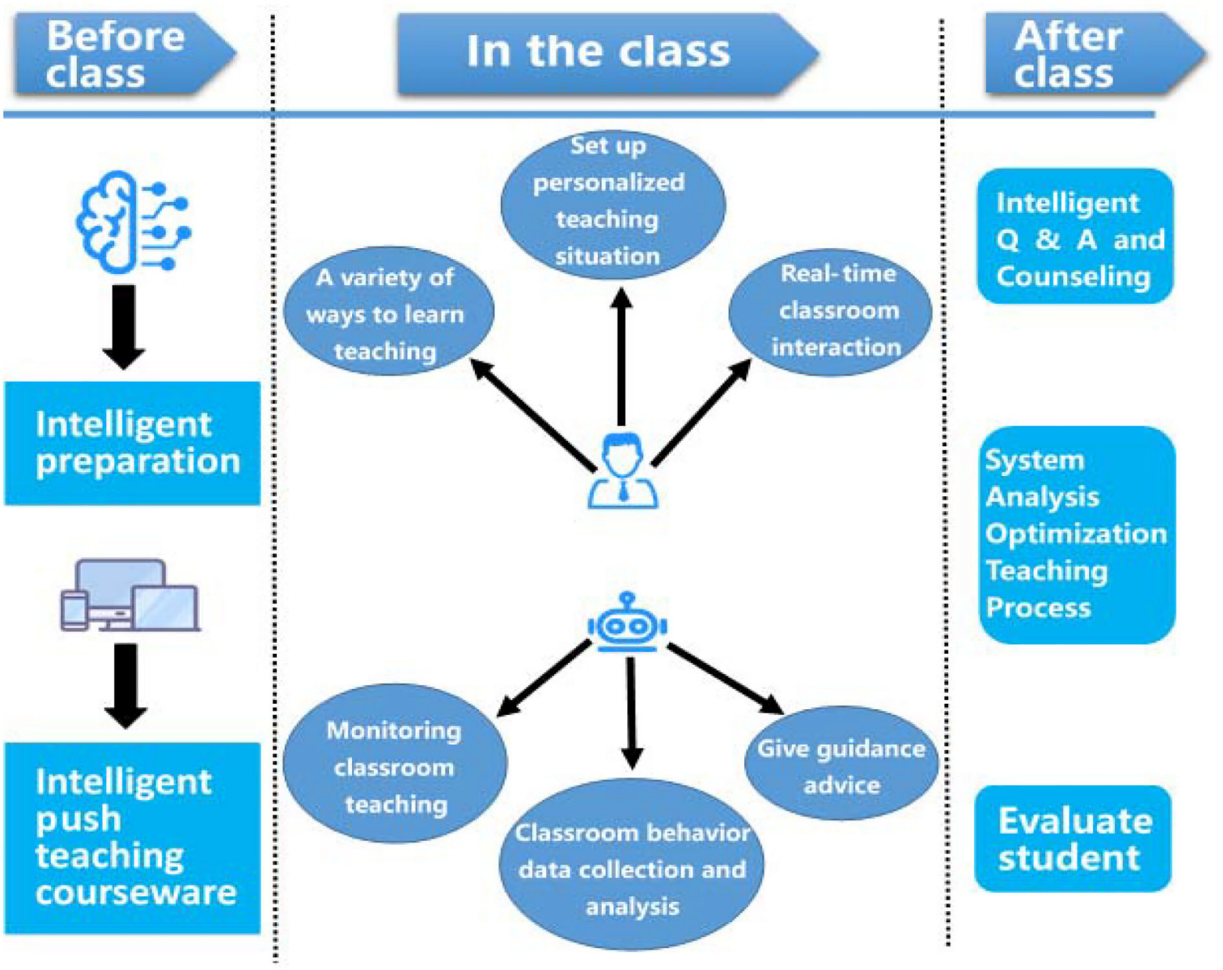
Research process of the proposed intelligent teaching model.

Image recognition has been widely used similar to neural networks, but CNN can only take one image as one input and one output at a time. There is no way to recognize multiple items at the same time, while You Only Look Once (YOLO) can recognize multiple objects at once. This study uses YOLOv3. YOLOv1 was proposed by Redmon, and Figure 2 is the network architecture diagram. Output an image with an input size of 448 × 448 as S × S grids (grid; the default is 7 × 7), and then detect whether there is an object in each grid separately, and generate B (default is 2) bounding boxes and N (default is 20) category of conditional class probabilities, the five prediction parameters in bounding boxes are x, y, w, h and confidence scores, x and y are the coordinates of the bounding boxes, w and h are the width and height of the bounding boxes, confidence scores is the value of whether there is an object 0 or 1, conditional class probabilities is the probability of N categories, and finally, the bounding boxes with the object are left, and then use Non-Maximum Suppression (NMS) to select the most suitable frame (Dai et al., 2020; Wang et al., 2021; Yu et al., 2021; Li et al., 2022; Zou et al., 2022).

The improvement content of YOLOv2 is to add anchor boxes, the output scale is 13 × 13 and each grid has 5 anchor boxes to predict bounding boxes, so the maximum output bounding boxes is 845, and the image input size is not limited to 448 × 448, but a multiple of 32 is the basic structure to change from the original GoogleNet to VGG-16, and Batch Normalization is added to prevent over-fitting and multi-scale training to improve the detection effect. The improvement content of YOLOv3 is to change the output scale to 13 × 13, 26 × 26, and 52 × 52, and each grid has 3 anchor boxes to predict bounding boxes. The Feature Pyramid Networks' multi-level prediction architecture improves the detection ability of small objects. The maximum number of output bounding boxes is 3,549, the loss function is changed from the original sum-squared error to binary cross-entropy, the output activation function is changed from the original softmax to logistic, and the basic architecture is changed from the original VGG-16 to ResNet (Cheng et al., 2019b; Zheng et al., 2019; Lu et al., 2020; Shen et al., 2021; Yu et al., 2021).

### SOLO taxonomy

The Structure of the Observed Learning Outcome (SOLO) taxonomy can be used to categorize student responses to open-ended questions. The rubric used to assess your ePortfolio is based on the SOLO taxonomy. SOLO illustrates the qualitative differences between student responses as it describes levels of understanding. It classifies outcomes in terms of their complexity, so that a judgment may be made on the quality of student responses to assessment tassifies understanding into five (5) levels (Essid et al., 2006; Bhattacharyya et al., 2012; Karvounidis et al., 2019; Ladias et al., 2020; Shi et al., 2020; Li et al., 2022):

- Prestructural: at this level, the learner is missing the point.
- Unistructural: a response based on a single point.
- Multistructural: a response with multiple unrelated points.
- Relational: points presented in a logically related answer.
- Extended abstract: demonstrating an abstract and deep understanding through unexpected extension.

## Research method analysis

### Research method

We make full use of artificial intelligence hardware and software such as big data, the Internet, and cloud computing to design teaching strategies under AI technology, proposing a model covering pre-class, in-class, and after-class teaching, respectively, as shown in Figure 2. The first round of action research is designed to guide deep learning. The second round of action research stimulates deep learning. The third round of action research reinforces deep learning. The three rounds of action research were divided into five parts: planning, action implementation, observation and analysis, assignment collection, and reflection on problems. In-class teaching was conducted in three sessions: before, during, and after class.

#### Careful design before class

Learning tasks were carefully designed according to the objectives of teaching, and pre-class learning resources were distributed to students through the Intelligent Learning Partner Education Cloud Platform. Students could study independently, actively explore the tasks, and ask questions or raise doubts. Teachers conducted an intelligent analysis based on the feedback from the platform to accurately grasp the characteristics of students and the degree of students' understanding of knowledge. In this way, teachers could modify the design of the course with a problem-oriented approach, which laid the foundation for refined teaching in class. Pre-class tasks required students' independent completion and group work to improve students' understanding and mastery of knowledge and help them gradually construct a system of knowledge.

#### Refined teaching in class

Teachers introduced the topic in a contextualized way, reviewed the difficult knowledge points found before class, and guided students to explore the problems independently so that they could grasp the essence of the problems and master the key points in learning. After resolving the difficult points detected before class, teachers introduced new concepts and allowed students to boldly try to explore the sources and connotations of knowledge through brainstorming. During the whole process, teachers monitored students' completion of assignments in real time with the help of the terminal. Students uploaded their works to the platform for display and sharing, and teachers evaluated students' results of learning, giving precise illustrations to improve their system of knowledge. In this way, the cultivation and improvement of students' abilities and familiarization and internalization of knowledge could be achieved, which highlighted teacher–student interactions, collaborative inquiry, and real-time supervision.

#### Further tutoring after class

Teachers assigned open-ended and innovative questions to students based on their pre-class tests and in-class investigations to check for gaps and consolidate their system of knowledge. In this way, teachers helped students to summarize and extend theoretical knowledge to trigger positive emotions. After-class tasks were assigned individually according to students' conditions of learning, and teachers provided personalized online tutoring to guide students to reflect on and summarize their learning, leading to their improvement in a personalized way (Cheng et al., 2019a,b).

## Findings and discussion

### Analysis of students' works

According to the theory of SOLO taxonomy in the evaluation of deep learning, and in combination with the deep learning assessment method, after each round of action research, we collected the personal resume works of 40 students in the experimental class and control class (experimental class: intelligent in-class teaching model; control class: traditional in-class teaching model) and classified students' works according to the general rubrics of grading. Students' works were divided into several levels according to the theory of SOLO taxonomy to evaluate understanding. The results in Table 1 were obtained by counting the number of students' works in each level in the experimental class (Class A) and control class (Class B).

**Table 1:** The number of students' works in each level of understanding.

	**Group**	**PS**	**US**	**MS**	**R**	**EA**
First round of action research	A	11	13	12	4	0
	B	12	11	13	4	0
Second round of action research	A	6	13	11	9	1
	B	7	15	12	6	0
Third round of action research	A	0	7	16	13	4
	B	4	9	19	8	0

PS, pre-structural level; US, unistructural level; MS, multistructural level; R, relational level; EA, extended abstract level.

Through an analysis of Table 1, it can be seen that only four students in both the experimental and control classes were in deep learning in the first round of action research, while most of the students were in a state of superficial learning or no learning. In general, at the beginning of the experiment, there was little difference in the cognitive level between the experimental and control classes. In the second round of action research, 10 students in the experimental class achieved deep learning, and the number of students in the state of no learning decreased compared to the first round. However, students in the state of superficial learning were still the majority. In contrast, the number of students who achieved deep learning in the control class was six, which was smaller than that in the experimental class. In the third round of action research, the number of students who achieved deep learning in the experimental class reached 17. Among these 17 students, four students' works were at the level of extended abstract structure, while eight students in the control class reached the state of deep learning, which was fewer compared to the experimental class. In this round of action research, there was no student in the experimental class in the pre-structured state of learning, while there were four students in the control class. This shows that students' state of learning was constantly improving and that teaching and learning under the intelligent in-class teaching model was more effective (Bhattacharyya et al., 2012; Shi et al., 2020).

**Figure 3:**
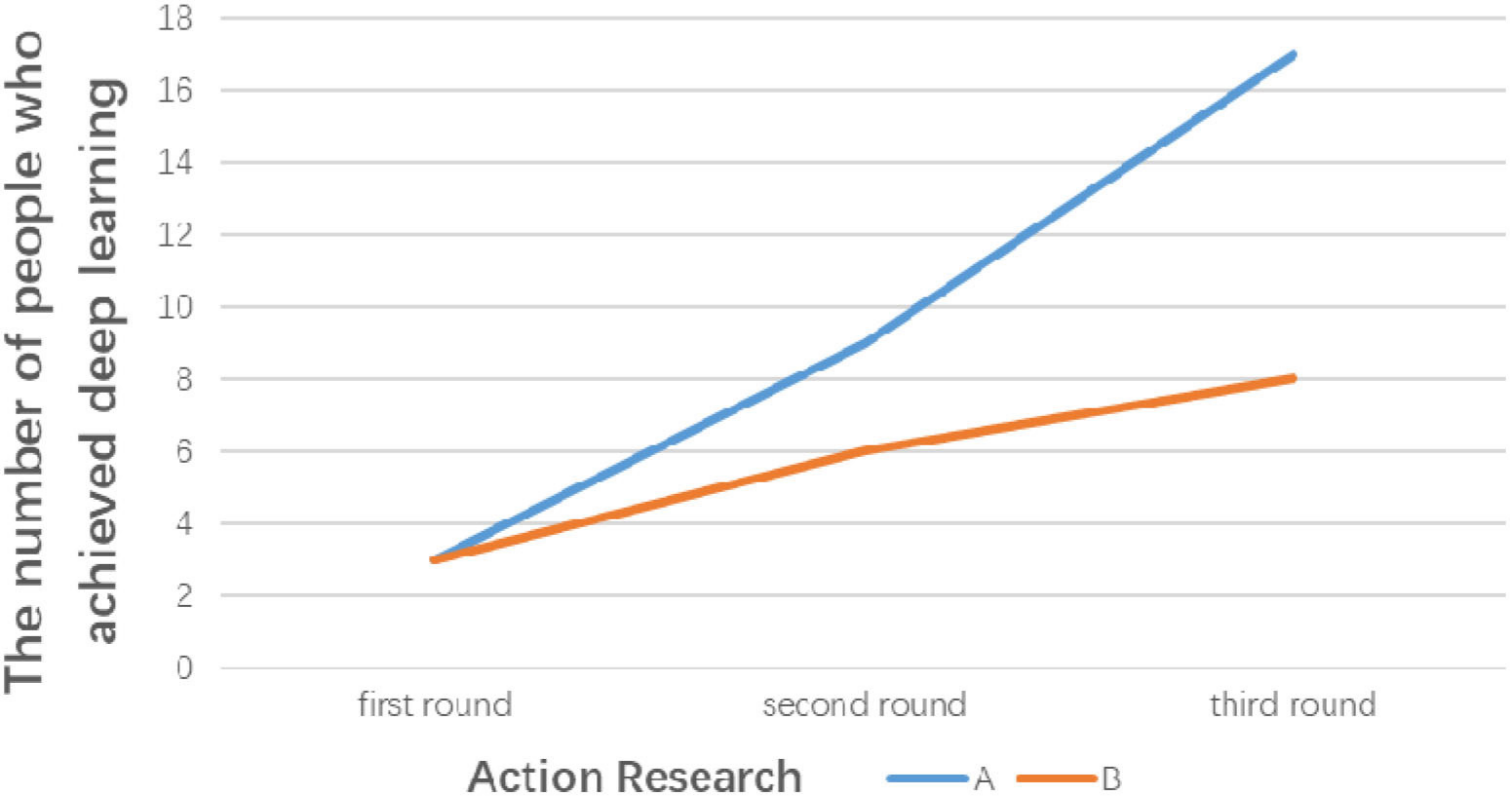
The number of people who achieved deep learning in the three rounds of action research.

A statistical analysis of the numbers of students who reached deep learning (i.e., R and EA) in the experimental class (Class A) and the control class (Class B) in the three rounds of action research is shown in Figure 3. The table clearly shows that the number of students in the experimental and control classes achieving deep learning in the first round of action research was the same; after the second and third rounds of action research, the number of students who achieved deep learning gradually increased. In comparison, however, the experimental class saw more students achieving deep learning than the control class. The analysis of the quality of students' works shows that the intelligent in-class teaching model can facilitate students' deep learning compared to the traditional teaching model.

### Finding students' scores

Before the implementation of the experimental action research, a basic test on personal resume design and production was conducted in the experimental and control classes to assess the initial state of the two classes in terms of knowledge before the upcoming implementation of the intelligent in-class teaching model. The results of the test were used as the pre-test data for evaluating students' mastery of knowledge. At the end of the three rounds of experimental action research, the experimental and control classes were tested again separately for the mastery of knowledge, and the test scores were used as the post-test data of students' mastery of knowledge. After the data collection was completed, we performed a statistical analysis of the students' pre-test and post-test scores and conducted independent samples *t*-test using SPSS 24.0. The results of the statistical analysis are shown in Table 2.

**Table 2:** Statistical analysis of students' pre-test and post-test scores.

	**Class**	**Number of** **cases**	**Mean**	**Standard** **deviation**	***t*-test**	**P**
Pre-test	Experimental class	40	52.50	20.196	−0.244	0.808
	Control class	40	53.61	18.385	−0.244	0.808
Post-test	Experimental class	40	73.75	13.752	4.456	0.000
	Control class	40	59.58	13.222	4.456	0.000

Pre-test: The Chi-square test was performed on the independent samples before conducting the independent samples *t*-test. F was 0.656, and sig was 0.421, which was >0.05. Therefore, the two samples passed the Chi-square test. The independent samples *t*-test could be conducted, where *p* was 0.808, which was >0.05, indicating that there was no significant difference between the scores of the two classes.

Post-test: The Chi-square test was performed on the independent samples before conducting the independent samples *t*-test. F was 0.002, and significance was 0.968, which was >0.05. Therefore, the two samples passed the Chi-square test. The independent sample *t*-test could be conducted, where *p* was 0.000, which was <0.05, indicating that there was a significant difference between the scores of the two classes. Furthermore, the mean of the experimental class was higher than that of the control class, indicating that the intelligent in-class teaching model was better than the traditional teaching model.

### Finding students' level of deep learning

To verify whether students' level of deep learning was enhanced through the intelligent in-class teaching model, we conducted a pre-test and post-test to examine students' state and ability of deep learning, respectively. First, in this study, we analyzed the level of deep learning of students in terms of two dimensions. One is to analyze students' state of deep learning, and the other is to analyze students' ability of deep learning. We tested the reliability and validity of the questionnaires from these two dimensions, respectively. We distributed questionnaires to a total of 82 persons, and 78 valid questionnaires were returned. We then conducted the validity test using SPSS24.0. In the reliability analysis, the Cronbach coefficient was 0.967, indicating high reliability. Reliability analysis was also conducted for each dimension separately, and the reliability of the Cronbach coefficient obtained for each dimension was >0.7 for both dimensions, indicating that the questionnaire has high reliability and can be formally put into use. The KMO value obtained was 0.890, which is >0.6. The *p*-value was 0.000, which is smaller than 0.05. The results show that this questionnaire has high validity and can be formally put into use. After the reliability and validity analysis, the pre-test and post-test data were analyzed to examine the level of deep learning of the students in the experimental group (using the intelligent in-class teaching model) and control group (traditional in-class teaching model) (Table 3).

**Table 3:** The independent sample's *t*-test of the pre-test and post-test for each dimension of the level in the two groups.

**Dimension**		**Group**	**Research** **sample**	**Mean**		**Standard deviation**		**Significance** **(two-tailed)**	
				**Pre-test**	**Post-test**	**Pre-test**	**Post-test**	**Pre-test**	**Post-test**
State of	Learning	Experimental class	40	**1.5764**	**1.5903**	**0.58499**	**0.44382**	0.288	0.013
deep learning	motivations	Control class	40	**1.7847**	**1.9722**	**1.00739**	**0.77639**		
	Learning	Experimental class	40	**2.0347**	**1.6875**	**0.82841**	**0.53577**	0.820	0.004
	inputs	Control class	40	**1.9861**	**2.1250**	**0.92274**	**0.92274**		
	Deep	Experimental class	40	**1.9861**	**1.4861**	**0.79044**	**0.40508**	0.323	0.007
	learning strategy	Control class	40	**2.2153**	**1.8264**	**1.13256**	**0.61766**		
Deep learning	Independent	Experimental class	40	**2.1019**	**1.6019**	**0.83121**	**0.54522**	0.648	0.004
ability	learning ability	Control class	40	**2.0000**	**2.0833**	**2.0833**	**0.81015**		
	Problem	Experimental class	40	**1.5833**	**1.5556**	**0.66368**	**0.61205**	0.091	0.035
	solving ability	Control class	40	**1.9537**	**1.8796**	**1.11028**	**0.66739**		
	Teamwork	Experimental class	40	**1.7317**	**1.4444**	**0.76353**	**0.45774**	0.410	0.001
	ability	Control class	40	**1.9259**	**1.9259**	**1.18217**	**0.66242**		
	Innovation	Experimental class	40	**1.6852**	**1.5278**	**0.71245**	**0.55990**	0.237	0.013
	ability	Control class	40	**1.9362**	**1.9362**	**1.9362**	**0.61284**		

As can be seen from Table 3, the standard deviations of the state and ability of deep learning of the experimental class were smaller than those of the control class, which indicates that the dispersion was lower. The analysis of the pre-test data by *t*-test reveals that the *p*-values were >0.05, indicating that there was no significant difference between the experimental class and the control class. The *p*-values of the state and ability of deep learning in the analysis of the post-test data were smaller than 0.05, indicating that there was a significant difference between the two groups on these two dimensions.

The comparison between the pre-test and post-test results indicates that there was no significant difference between the experimental class and control class regarding the state and ability of deep learning at the initial stage. After three rounds of action research, students in the experimental class generally chose “very satisfied” or “satisfied” on the two dimensions, which indicates that the intelligent in-class teaching model has helped students learn and effectively improve their level of deep learning.

### Analysis of students' emotional experience from interviews

After the implementation of the three rounds of action research, 15 students were randomly selected from the experimental class to participate in interviews and surveys. The results show that students preferred intelligent in-class teaching and believed that this model of teaching could help them improve all aspects of their abilities very fast, consolidate knowledge, exercise practical skills, and boost learning motivations and group inquiry skills.

Through the three rounds of action research, students' levels of knowledge and levels of deep learning have been improved, which indicates that in-class teaching under the intelligent teaching model can effectively promote students' abilities of deep learning. Because the whole course design is step-by-step and progressive, this model ultimately realized the promotion of deep learning and creates an intelligent and personalized learning atmosphere.

## Comparison and discussion

Through the analysis of the experimental data, we compared and analyzed the basic characteristics of traditional education, information-based education, and AI-based education, and we summarized the problems of teaching and learning in the application of AI technology in universities.

### Comparative analysis of traditional education and information-based education

The development of education has undergone two changes: the first is the change from traditional education to information-based education; the second is the change from information-based education to AI-based education. Table 4 shows the comparison between traditional education and informatization-based education. Traditional education is mainly teacher oriented, with limited teaching resources and a monotonous environment, which is not conducive to mobilizing students' motivations and has temporal and geographical restrictions. Informatization-based teaching is more student oriented with rich and diversified teaching resources, which makes full use of the advantages of modern pedagogical media, such as computers and the Internet, to boost students' enthusiasm. It is not limited by time and location, and the teaching and learning environment is also transitioning to Internet-based intelligent teaching and learning platforms.

**Table 4:** Analysis of the basic characteristics of traditional education and information-based education.

**Basic features**	**Traditional education**	**Information-based education**
Ways of teaching	Teacher-oriented, unified instruction	Student-oriented, mixed teaching and learning
Ways of learning	Listening to lectures **+** group study	Independent study based on a resource pool **+** digital campus
Teaching resources	Textbook + PPT + video + blackboard, etc.	Computer **+** Resource pool **+** Internet **+V** R ^**+**^ **A** R
Teaching environment	Classroom **+** Lab	Flipped classroom **+** Rain classroom, etc.

**Figure 4:**
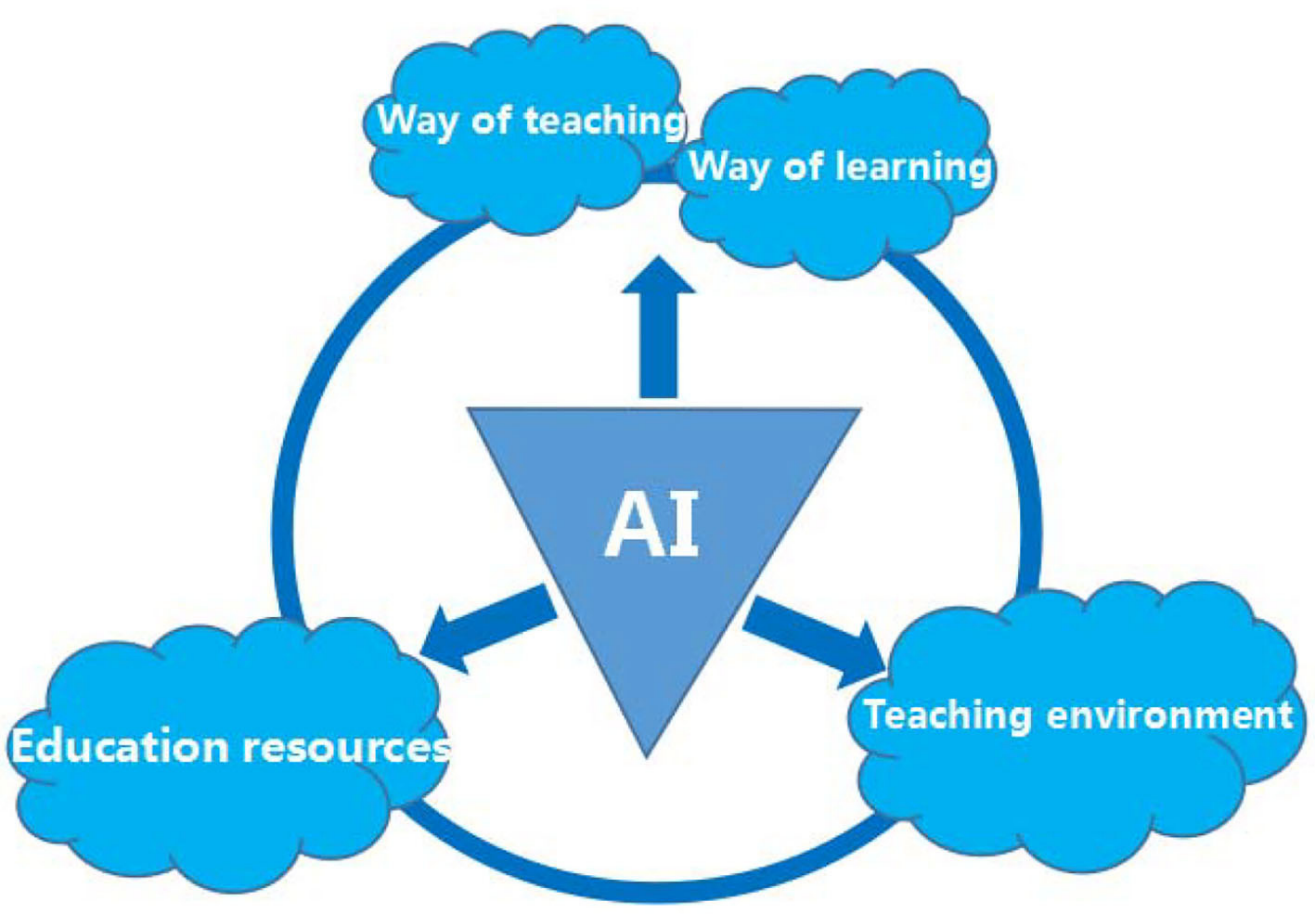
Framework of artificial intelligence technology for teaching and learning reform.

Although information-based teaching has played an indispensable role in the development of education and pedagogy, it overemphasizes the use of shared resource pools and modern information technology, while teachers might not be adequately informed of students' mastery of knowledge and students cannot independently choose modules of their interests to learn. In addition, certain knowledge has some constraints and cannot be practically demonstrated under information-based teaching. However, in the artificial intelligence environment, technology is not only used as a tool or means but has broken through the limitations of the previous role of technology, striving to achieve the integration of the objectives, content, and environment of teaching and other elements of the system with information technology. The overall framework of artificial intelligence for teaching and learning reform is shown in Figure 4.

### Discussion of the problems of teaching and learning under artificial intelligence technology

Currently, there are two main problems to discuss for university teachers and students to engage in AI technology-based teaching and learning, which are detailed as follows.

#### Lack of teacher–student interaction

Most of the current AI courseware relies on self-learning and operation by students, making it difficult for teachers to fully grasp the conditions of students. When students encounter problems, they cannot seek help from teachers in time. Teachers and students are sealed off from each other and cannot interact with each other. This situation greatly reduces the effectiveness of the courseware. There are still many unreasonable aspects in the teaching process, with an over-emphasis on the teachers' instruction and relatively little time for students' independent thinking.

#### Lack of human–computer interaction

Most existing AI uses a CD-ROM as the carrier of information to display the content of the teaching materials. It provides the information taught to the students mechanically according to pre-defined teaching processes, and students are completely passive in using the AI courseware for learning. Moreover, in the actual teaching process, teachers can only operate according to the pre-designed processes of the courseware, and human–computer interaction has not been adequately realized.

#### Building an innovative teaching model based on artificial intelligence technology

Designing an innovative teaching model based on AI technology: Aiming at teachers, students, and machines as the primary subjects, we make full use of artificial intelligence hardware and software such as big data, the Internet, and cloud computing to design teaching strategies under AI technology, proposing a model covering pre-class, in-class, and after-class teaching, respectively. The teaching methods under this model can achieve precision and personalized teaching through human–machine integration and intelligent real-time interaction between teachers and students with discussion as follows (Essid et al., 2006; Bhattacharyya et al., 2012; Karvounidis et al., 2019; Ladias et al., 2019, 2020; Shi et al., 2020).

##### Intelligent lesson preparation

Lesson preparation is the basis for teachers to carry out teaching. However, most teachers analyze students' conditions of learning based on their subjective awareness and experiences and often use teaching materials and courseware from ready-made Internet resources, which makes it difficult to grasp the teaching schedule and objectives. Therefore, to overcome these shortcomings, this article proposes intelligent lesson preparation using the lesson planning software Waston. This software can record data about students' learning conditions in real time. Through a big data intelligent analysis, it can push various high-quality teaching resources to teachers according to the knowledge points in specific chapters in line with the analysis of learning conditions and push personalized courseware to students.

##### In-class personalized teaching

The current information-based teaching model has proved to face obstacles in improving the quality of teaching and boosting students' motivations. AI technology, in contrast, can solve this problem well. We can use the Knewton platform to dynamically design a combination of personalized content according to students' preferred styles of learning, use a variety of methods to simulate situations for teaching and learning, and adopt multi-screen switching and interactions as well as in-class quiz and commenting to fully mobilize students' enthusiasm in learning. In addition, we can use image processing technology and speech recognition technology in AI technology to analyze and process videos recorded in the classroom. Through the technical processing of videos and recordings, teachers can grasp the dynamics of each student in the classroom, discover the problems in time, and carry out targeted teaching to truly realize personalized and humanized teaching.

##### After-class intelligent Q&A tutoring and optimization of pedagogical activities

Due to the limited time left for teachers to answer questions in the classroom, some students' questions cannot be addressed in class. We have designed a corresponding plan to solve this problem. First, the Waston intelligent system has established an expert knowledge pool, which allows intelligent online guidance for answering questions. For more typical questions, the system will forward them to teachers. After teachers answer the questions and upload the answers, they will be automatically sent to the students. Second, tutoring can be conducted with the help of AR technology. First, students set the camera of their mobile phone to AR mode. Then, students move the camera lens to the knowledge point that they do not understand. After matching and analyzing, it will be converted into a 3D model knowledge point, which is visualized and easy to understand, greatly enhancing the learning ability of students. For students, AI technology can act as a tutor to analyze each student's knowledge foundation and styles of interest, offer learning courseware to students that suits their traits, and then provide feedback to the instructors on the results. Second, AI technology can act as a companion to learning for students and create a reasonable learning plan for them, accompany the exercises, and monitor them to complete the exercises within the specified time limit. Third, AI technology can also play the role of students' playmates. After completing the learning tasks, AI assistants can accompany students to do exercises, sing, and so on, so that students can relieve themselves from the stress of learning.

Designing a learning model for students based on artificial intelligence technology: Currently, the learning model of most students is to listen to lectures and read textbooks, calculate on paper, and complete after-class assignments. Each student performs the same learning tasks and activities, which are boring and tedious. The mode of assessment is focused on testing the grasp of knowledge points. Such problems can be effectively solved by AI-assisted learning for students.

##### Students learn adaptively before class

Knewton's adaptive platform can provide pre-class learning materials that are tailored to each student's abilities and interests. Although the materials provided are slightly different, the principles behind the adaptive content are the same. When students encounter difficulties, the system automatically reduces the difficulty of the content, that is, it constantly adjusts the students' content of learning according to their performance in the learning process, which provides a personalized learning experience that makes learning easier and more efficient for students.

##### Students learn through human–computer interaction and group interaction in class

In class, the intelligent system and platform provide students with personalized learning services, and students can use their mobile phones to complete interactive tasks, such as check-in, raising questions, answering questions, completing exercises, etc., which is conducive to a lively classroom atmosphere and efficient learning. The intelligent teaching platform can also reasonably group students according to their strengths, realize interactive learning among groups, and let students work and discuss together to complete the learning tasks assigned by teachers, which is conducive to cultivating students' ability to analyze and solve problems and work in teams.

##### Students carry out personalized review after class

With AI technology acting as a tutor, it can answer questions for students at any time. As a learning companion, on the one hand, AI technology can supervise students to complete their learning tasks within the specified time limit, praise students if they finish on time, and urge and reprove them if they do not finish on time. On the other hand, students can check teachers' evaluations of them on the mobile phone terminal, reflect on their conditions of learning, and make up for their shortcomings.

## Conclusions and future work

This article has the following contributions:

(1) Changing the subject of teaching and learning: For teachers, AI technology can be used as an assistant to help teachers with intelligent lesson preparation, answering questions, and grading assignments, which provides teachers with more energy and time to know each student better. Second, AI technology can act as a companion to learning, indicating the problems that teachers face during the teaching process at any time and giving suggestions for improving teaching. Third, AI technology can also act as a virtual student to help teachers practice in-class teaching in advance or prepare for demonstration teaching.
(2) Designing an innovative teaching model based on artificial intelligence technology: Aiming at teachers, students, and machines as the primary subjects, we make full use of artificial intelligence hardware and software such as big data, Internet, and cloud computing to design teaching strategies under AI technology, proposing a model covering pre-class, in-class, and after-class teaching, respectively.
(3) Designing a learning model for students based on artificial intelligence technology: Reduce the learning model of most students is to listen to lectures and read textbooks, calculate on paper, and complete after-class assignments. Each student performs the same learning tasks and activities, which are boring and tedious. The mode of assessment is focused on testing the grasp of knowledge points.

In the future work, we need more data on experimental study that reflect the situation of university teachers and students in applying AI technology for teaching and learning in a relatively objective and realistic way. However, there are still certain limitations mainly because the scope and targets of this study are not broad enough. In the future, we will further expand the scope of the survey and research to obtain more first-hand information and discover more problems and factors affecting the application of AI technology for education and pedagogy to provide references for improving the quality of teaching.

## Data availability statement

The original contributions presented in the study are included in the article/supplementary material, further inquiries can be directed to the corresponding author.

## Ethics statement

Ethical review and approval was not required for the study on human participants in accordance with the local legislation and institutional requirements. Written informed consent from the patients/participants or patients/participants' legal guardian/next of kin was not required to participate in this study in accordance with the national legislation and the institutional requirements.

## Author contributions

YL contributed to the work concept or design and data collection. LC and ZY drafted the manuscript and make an important revisions to the manuscript. All authors approved the final version of the manuscript for publication.

## Conflict of interest

The authors declare that the research was conducted in the absence of any commercial or financial relationships that could be construed as a potential conflict of interest.

## Publisher's note

All claims expressed in this article are solely those of the authors and do not necessarily represent those of their affiliated organizations, or those of the publisher, the editors and the reviewers. Any product that may be evaluated in this article, or claim that may be made by its manufacturer, is not guaranteed or endorsed by the publisher.

## Appendix

### Questionnaire content

1. For teachers, AI technology can be used as an assistant to help them with intelligent lesson preparation, answering questions, and grading assignments?
2. Based on artificial intelligence technology for teachers, students, and machines as the primary subjects, the use of artificial intelligence hardware and software such as big data, the Internet, and cloud computing to design teaching strategies under AI technology, proposes a model covering pre-class, in-class, and after-class teaching, respectively?
3. A learning model for students based on artificial intelligence technology, where most students listen to lectures and read textbooks, calculate on paper, and complete after-class assignments. Each student performs the same learning tasks and activities, which are boring and tedious. The mode of assessment is focused on testing the grasp of knowledge points?
4. With AI technology acting as a tutor, it can answer questions for students at any time?
5. In class, the intelligent system and platform provide students with personalized learning services, and students can use their mobile phones to complete interactive tasks, such as check-in, raising questions, answering questions, and completing exercises?
6. AI is an effective way to enhance students' motivation and active learning?
7. AI can improve teachers' syllabus and teaching material content (including digital teaching materials) Internet access rate?
8. AI can enhance students' creativity and innovation in course content and the effectiveness of project production?
9. AI can improve the teaching quality control mechanism and track the improvement of teaching evaluation, the specific results, and unique and innovative plans to improve teaching effectiveness?
10. AI can enhance the planning of teaching characteristics, shape respect, and promote teaching and learning?
11. The psychological feeling and influence that AI technology can bring to people vary according to the user's preferences, habits, and acceptance?
12. AI technology can learn from massive data through the system, and then correctly interpret external data, and use this knowledge to achieve specific goals and tasks?
